# Factors influencing early postoperative blood pressure goal achievement after endovascular repair for type B aortic dissection: development of a refined nursing monitoring protocol

**DOI:** 10.3389/fcvm.2026.1777778

**Published:** 2026-04-17

**Authors:** Dongmei Xu, Chen Chen, Zhen Wan, Tao Zhou

**Affiliations:** Department of Cardiothoracic Surgery, Nanjing Hospital, Nanjing Medical University (Nanjing First Hospital), Nanjing, Jiangsu, China

**Keywords:** endovascular repair, monitoring protocol, refined nursing, time to bloodpressure goal achievement, type b aortic dissection

## Abstract

**Objective:**

To investigate the factors influencing the time to achieve early postoperative blood pressure (BP) goals in patients with type B aortic dissection (TBAD) following thoracic endovascular aortic repair (TEVAR) and to develop a refined nursing monitoring protocol.

**Methods:**

A retrospective cohort study was conducted, consecutively enrolling 142 TBAD patients who underwent TEVAR. Baseline patient characteristics, surgery-related indices, and postoperative indices were collected, with the primary observational endpoint being the time to achieve early postoperative BP goals. Univariate analysis and Cox regression analysis were employed to identify influencing factors. Based on the analytical results and combined with evidence-based practices, a refined nursing monitoring protocol was developed and refined using the Delphi expert consultation method.

**Results:**

The median time to achieve early postoperative BP goals among the 142 patients was 9.5 h (interquartile range: 6.0–13.8 h). Multivariate Cox regression analysis revealed that advanced age (HR = 0.97, *P* = 0.011), history of hypertension (HR = 0.54, *P* = 0.005), higher postoperative initial systolic BP (SBP) (HR = 0.976, *P* = 0.003), and elevated postoperative pain score (HR = 0.77, *P* = 0.002) were associated with a slower rate of achieving BP goals, thus identified as independent factors for prolonging the time to BP goal achievement.

**Conclusion:**

Advanced age, history of hypertension, higher postoperative initial SBP, and postoperative pain are independent factors prolonging the time to achieve BP goals in TBAD patients after TEVAR. The refined nursing monitoring protocol developed from these findings is targeted, systematic, and practical, providing an evidence-based framework for optimizing postoperative management, which holds potential for improving patient prognosis and warrants prospective validation.

## 1 Introduction

Type B aortic dissection (TBAD) is a critical and acute condition primarily caused by a tear in the aortic intima, allowing blood to enter the aortic wall and create a false lumen. It manifests abruptly with a severe clinical course and is associated with high rates of mortality and morbidity. Epidemiological studies indicate an annual TBAD incidence of approximately 2.9 to 4.7 cases per 100,000 individuals, with higher susceptibility in males and the elderly, often accompanied by underlying conditions such as hypertension and atherosclerosis (1). Without timely intervention, patients may succumb to severe complications including aortic rupture and malperfusion syndrome. With advancements in endovascular techniques, thoracic endovascular aortic repair (TEVAR) has become a first-line treatment for complicated TBAD. TEVAR involves implanting a stent-graft to cover the primary entry tear, promoting false lumen thrombosis and aortic remodeling, thereby significantly reducing the risk of aortic-related mortality (2, 3). However, TEVAR is still associated with various complications such as endoleak, stent-graft migration, and continued aortic expansion, which adversely affect patient outcomes (2, 4, 5).

Blood pressure (BP) control is considered a pivotal aspect of post-TEVAR management. Strict BP management not only helps reduce aortic wall stress, minimizing stent-graft-related complications, but also improves organ perfusion and facilitates false lumen closure and aortic remodeling (6–8). The time to achieve early postoperative BP goals (i.e., the duration from surgery completion until BP first reaches the target range) is a crucial indicator for assessing hemodynamic stability. Delayed achievement may increase the risk of postoperative complications, prolong hospital stay, and impede patient recovery (9–11). Recent studies suggest that early and strict BP control after TEVAR is associated with a reduced risk of endoleak, enhanced false lumen thrombosis, and favorable aortic remodeling, which are key determinants of long-term prognosis (12–15). Consequently, the “time to achieve BP goals” serves as a quantifiable process quality indicator. Its shortening may signify a smoother postoperative course and better anatomical outcomes, underscoring its clinical relevance as an intermediary endpoint (16).

Currently, while some domestic and international studies have explored strategies for post-TEVAR BP management, there remains a lack of systematic analysis regarding factors influencing the time to early BP goal achievement. Furthermore, existing nursing monitoring protocols require refinement in terms of precision and individualization. The primary shortcomings lie in the fact that current nursing measures often focus on monitoring single indicators, lacking a systematic and individualized monitoring framework based on multifactorial risk stratification. Additionally, the absence of a unified, standardized monitoring process leads to variability and randomness in clinical practice. Moreover, existing research provides insufficient comprehensive analysis of the multifactorial influences on this key process indicator, early BP goal achievement time, and fails to effectively translate evidence into structured, actionable nursing pathways.

Therefore, this study hypothesizes that patient-specific characteristics and surgery-related factors independently influence the time required for postoperative BP control. Accordingly, this study aims to systematically analyze the factors influencing the time to achieve early postoperative BP goals in TBAD patients after TEVAR via a retrospective cohort study. Based on these findings, a scientific and systematic refined nursing monitoring protocol will be developed to provide a theoretical basis and practical guidance for implementing precise nursing interventions in clinical settings.

## 2 Materials and methods

### 2.1 Study design

This retrospective cohort study consecutively enrolled patients diagnosed with TBAD who underwent TEVAR at our Hospital between January 2022 and December 2024. By systematically reviewing electronic medical records (EMRs), surgical records, and nursing records, relevant clinical data were collected to analyze factors influencing the time to achieve early postoperative BP goals and to construct a refined nursing monitoring protocol. This study was approved by the Ethics Committee of Nanjing Hospital, Nanjing Medical University (Nanjing First Hospital) (Approval No.: 202507182).

### 2.2 Study participants

#### 2.2.1 Inclusion and exclusion criteria

Inclusion criteria: (1) Age ≥18 years; (2) Diagnosed with acute (onset time ≤ 14 days) or subacute (onset time of 15 ∼ 90 days) Stanford TBAD based on clinical symptoms and computed tomography angiography, with diagnosis and staging criteria referring to the 2022 ESC Guidelines on the Diagnosis and Treatment of Aortic Diseases and the International Registry of Acute Aortic Dissection criteria (1, 17); (3) Undergoing TEVAR for the first time; (4) Complete clinical medical records, surgical records, and postoperative monitoring data.

Exclusion criteria: (1) Concurrent Stanford type A aortic dissection or other non-dissective thoracic aortic diseases (e.g., aneurysm); (2) History of prior aortic surgery or endovascular repair; (3) Pregnant or lactating women; (4) Intraoperative occurrence of severe complications leading to extreme hemodynamic instability; (5) Death or automatic discharge within 24 h postoperatively, preventing acquisition of valid BP goal achievement data; (6) Missing clinical data or incomplete recording of key data.

#### 2.2.2 Study sample

**Figure 1:**
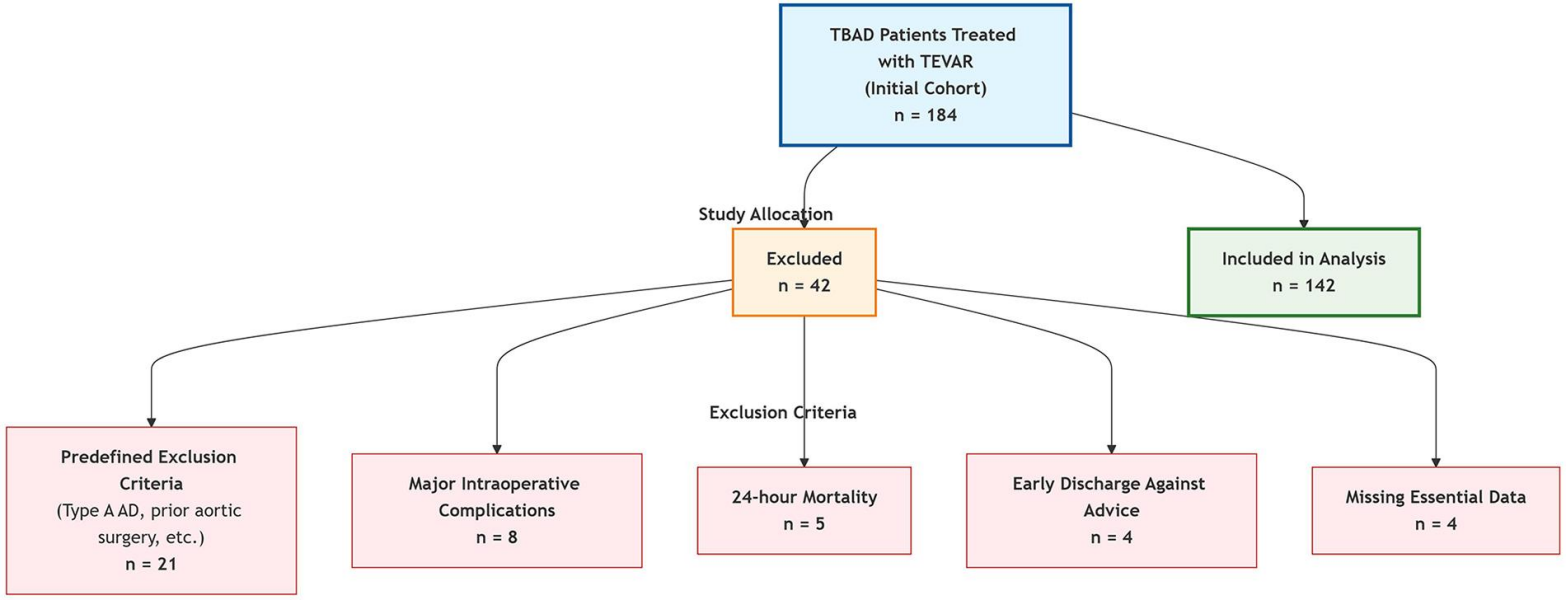
The CONSORT flow diagram.

The primary analysis employed a Cox proportional hazards regression model. As all enrolled patients were expected to achieve the BP goal (event), ensuring an adequate number of events per predictor variable was crucial. Based on the rule of thumb requiring at least 10 events per variable for Cox regression, and anticipating approximately 10 candidate variables for initial screening, a minimum sample size of 100 events was estimated. Ultimately, 142 patients (i.e., 142 events) were enrolled, which provided sufficient statistical power for the final multivariate model containing 4 variables.By reviewing the EMR system, 184 patients initially meeting the inclusion criteria who underwent TEVAR for TBAD at our Hospital between January 2022 and December 2024 were identified. After applying the inclusion and exclusion criteria, 42 patients were excluded. The detailed reasons for exclusion are presented in Figure 1 (STROBE flow diagram). Consequently, 142 patients were ultimately included in the analysis.

### 2.3 Research methods

#### 2.3.1 Study variables

This retrospective study collected the following variables by reviewing the hospital EMR system, anesthesia records, and monitoring records:

1. Time to achieve early BP goals: Defined as the duration from the end of the endovascular procedure until the patient’s BP first reached the target range [systolic BP (SBP): 100–120 mmHg; mean arterial pressure (MAP): 60–80 mmHg] simultaneously and was maintained for ≥ 30 min (18). This dual-target range aligns with contemporary guideline recommendations for managing acute aortic syndromes, aiming to balance organ perfusion with reduction of aortic wall stress (19–22).
2. Baseline data: Age, sex, body mass index (BMI), smoking history, alcohol consumption history, history of hypertension, comorbidities, etc.
3. Surgery-related indices: Operative time, anesthetic type, intraoperative blood loss, use of vasoactive drugs, TEVAR-related complications.
4. Postoperative indices: Initial postoperative BP, heart rate, pain score, serum creatinine, white blood cell count, C-reactive protein, frequency of BP monitoring, postoperative complications.

#### 2.3.2 Data collection methods

A standardized medical record information collection form was used. The time to achieve early BP goals was extracted and calculated from continuous BP monitoring records in intensive care unit (ICU) flow sheets and nursing records. Baseline data were obtained from admission records, initial nursing assessment forms, and past medical history. Surgery-related indices were retrieved from anesthesia records, surgical nursing records, and immediate postoperative imaging reports. Initial postoperative BP, heart rate, pain score [assessed using the Numerical Rating Scale (NRS), range 0–10, where 0 indicates no pain and higher scores indicate more severe pain], and frequency of BP monitoring were obtained from ICU flow sheets and nursing records. Laboratory indices (creatinine, white blood cell count, C-reactive protein, etc.) were retrieved from the laboratory information system for the first test result within 24 h postoperatively. Postoperative complications were confirmed and recorded by comprehensively reviewing progress notes, nursing records, consultation notes, and relevant auxiliary examination reports. All data were independently collected by two researchers and cross-checked to ensure accuracy and completeness. For continuous variables, the intraclass correlation coefficient (ICC) was used to assess inter-rater reliability. For categorical variables, the Kappa coefficient was used. A random re-measurement of 10% of the cases showed ICC values >0.85 and Kappa coefficients >0.75, indicating good inter-rater agreement. Missing values were assessed and handled after data collection. Cases with missing key variables were excluded.

### 2.4 Development process of the refined nursing monitoring protocol

Based on the results of the aforementioned factor analysis and incorporating evidence-based medicine methods, a refined nursing monitoring protocol was developed. A systematic search was conducted in the following databases: China National Knowledge Infrastructure, Wanfang, VIP, PubMed, Embase, Cochrane Library, and Web of Science, covering the period from January 2013 to December 2023. Search terms included aortic dissection, type B, endovascular repair, TEVAR, postoperative care, BP management, pain management, nursing, guideline, consensus. A combination of subject headings and free-text terms was used, and references of relevant articles were reviewed. Literature inclusion criteria were: ① Study population comprising adult TBAD patients undergoing TEVAR; ② Content related to guidelines, expert consensus, systematic reviews, or high-quality original research on postoperative BP management, pain management, complication monitoring, or nursing protocols. Exclusion criteria were: ① Duplicate publications; ② Unavailable full-text articles; ③ Reports with poor methodological quality; ④ Literature related to non-dissective aortic diseases. Through this search strategy, 18 relevant articles were initially retrieved. Ultimately, 4 articles were included as the evidence base for protocol development (23–25). The preliminary protocol content was formed by integrating this evidence. The Delphi expert consultation method was then used to revise and refine the preliminary protocol.

Fifteen experts were invited to form an expert panel, comprising 5 chief physicians of vascular surgery, 4 head nurses of cardiothoracic and vascular surgery, 3 ICU medical team leaders, and 3 quality management experts from the nursing department. The average work experience of the experts was (18.6 ± 5.3) years, all holding associate senior titles or higher. Two rounds of expert consultation were conducted. The first round solicited opinions on the protocol structure and specific items for modification. The second round confirmed and adjusted the revised details (e.g., monitoring time points, medication dosage ranges). The expert response rates for the two rounds were 100% and 93.3%, respectively. The expert authority coefficients were 0.87 and 0.89, respectively. The Kendall's W coefficients were 0.423 and 0.486 (both *P* < 0.01), indicating a high degree of expert opinion coordination and reliable results. Consensus was finally reached based on expert feedback, establishing the final refined nursing monitoring protocol.

### 2.5 Statistical analysis

Data processing and analysis were performed using SPSS 26.0 software. Normally distributed continuous data were presented as mean ± standard deviation (x¯ ± s), with inter-group comparisons using independent samples *t*-tests. Non-normally distributed continuous data were presented as median (interquartile range) [M (IQR)], with inter-group comparisons using the Mann–Whitney *U*-test. Categorical data were presented as number (percentage) [*n* (%)], with inter-group comparisons using the chi-square (*χ²*) test. All potential influencing factors were included in univariate analysis, with postoperative achievement of early BP goals as the endpoint event. Variables with *P* < 0.05 in univariate analysis were included in a Cox proportional hazards regression model. Before constructing the multivariate model, collinearity diagnostics were performed on the included variables, and all variance inflation factors were less than 2, indicating no severe multicollinearity. The proportional hazards assumption was tested using the Schoenfeld residuals method, and results showed *P*-values > 0.05 for all variables, satisfying the assumption. The final model analyzed the influence of each factor on the speed of BP goal achievement, where hazard ratio (HR) < 1 indicated that the factor was associated with a slower rate of achieving the BP goal (i.e., a risk factor for delayed achievement), and HR > 1 indicated a faster rate of achievement.All statistical tests were two-sided, with *P* < 0.05 considered statistically significant.

## 3 Results

### 3.1 Analysis of influencing factors

#### 3.1.1 Description of patient baseline characteristics

The baseline characteristics of the 142 TBAD patients who underwent TEVAR are shown in Table 1.

**Table 1:** Patient baseline characteristics.

Characteristic	Value
Demographics	
Age (years)	58.9 ± 11.2
Male [n (%)]	108 (76.1)
BMI (kg/m²)	26.0 ± 2.1
Smoking history [n (%)]	81 (57.0)
Alcohol history [n (%)]	72 (50.7)
Comorbidities [n (%)]	
Hypertension	112 (78.9)
Diabetes mellitus	25 (17.6)
Coronary artery disease	19 (13.4)
Chronic kidney disease	11 (7.7)
Cerebrovascular disease	16 (11.3)
Surgery-related indices	
Operative time (min)	182.3 ± 26.1
Intraoperative blood loss (mL)	340 [210, 510]
Intraoperative vasoactive drug use [n (%)]	126 (88.7)
TEVAR-related complications [n (%)]	18 (12.7)
Postoperative complications [n (%)]	16 (11.3)
Time to early BP goal achievement (h)	
Range	3.5–28.0
M (IQR)	9.5 (6.0, 13.8)
x¯ ± s	11.5 ± 6.8
Distribution of achievement time [n (%)]	
≤6 h	36 (18.3)
>6 and ≤12 h	54 (38.0)
>12 and ≤24 h	41 (28.9)
>24 h	11 (7.7)

BMI, body mass index; TEVAR, Thoracic endovascular aortic repair; BP, Blood pressure; *n* (%), Number (percentage); M (IQR), Median (interquartile range); x¯ ± s, Mean ± standard deviation.

#### 3.1.2 Univariate analysis of factors influencing time to BP goal achievement

To explore factors influencing the time to achieve early BP goals after TEVAR for TBAD, patients were divided into a rapid achievement group (≤9.5 h, *n* = 71) and a slow achievement group (> 9.5 h, *n* = 71) based on the median achievement time (9.5 h). Univariate analysis showed that patients in the rapid achievement group were younger, had less intraoperative blood loss, a lower prevalence of hypertension, and lower postoperative initial SBP and pain scores. Differences between groups were statistically significant (*P* < 0.05) Table 2.

**Table 2:** Univariate analysis of factors influencing time to BP goal achievement.

Variable	Rapid achievement group (*n* = 71)	Slow achievement group (*n* = 71)	Statistic	*P*
Demographics				
Age (years)	55.3 ± 10.1	60.5 ± 11.6	*t* = −2.89	0.005
Male [n (%)]	55 (77.5)	53 (74.6)	*χ²* = 0.16	0.689
BMI (kg/m²)	25.2 ± 3.1	26.0 ± 3.6	*t* = −1.43	0.155
Smoking history [n (%)]	38 (53.5)	43 (60.6)	*χ²* = 0.74	0.390
Alcohol history [n (%)]	34 (47.9)	38 (53.5)	*χ²* = 0.46	0.498
Comorbidities [n (%)]				
Hypertension	50 (70.4)	62 (87.3)	*χ²* = 6.31	0.012
Diabetes mellitus	10 (14.1)	15 (21.1)	*χ²* = 1.21	0.272
Coronary artery disease	8 (11.3)	11 (15.5)	*χ²* = 0.56	0.454
Chronic kidney disease	4 (5.6)	7 (9.9)	*χ²* = 0.845	0.357
Cerebrovascular disease	6 (8.5)	10 (14.1)	*χ²* = 1.12	0.290
Surgery-related indices				
Operative time (min)	175.6 ± 40.2	189.2 ± 46.1	*t* = −1.89	0.061
Intraoperative blood loss (mL)	320 (195, 480)	360 (230, 540)	*Z* = −1.97	0.049
Vasoactive drug use [n (%)]	60 (84.5)	66 (93.0)	*χ²* = 2.75	0.097
TEVAR-related complications [n (%)]	7 (9.9)	11 (15.5)	*χ²* = 1.04	0.308
Postoperative indices				
Postoperative initial SBP (mmHg)	138.5 ± 12.3	148.2 ± 14.6	*t* = −4.28	<0.001
Postoperative initial heart rate (bpm)	76.8 ± 8.5	79.3 ± 9.1	*t* = −1.68	0.095
Postoperative pain score (points)	3.2 ± 1.1	4.1 ± 1.3	*t* = −4.52	<0.001

BP, Blood pressure; BMI, Body mass index; TEVAR, Thoracic endovascular aortic repair; SBP, Systolic blood pressure.

### 3.13 Multivariate Cox regression analysis of factors influencing time to BP goal achievement

Multivariate analysis was performed using a Cox proportional hazards regression model. Using the achievement of early postoperative BP goals as the endpoint event, variables with *P* < 0.05 in the univariate analysis were included in the multivariate Cox regression model. The results showed that advanced age, history of hypertension, higher postoperative initial SBP, and higher postoperative pain score were independent risk factors for delaying postoperative BP goal achievement (all *P* < 0.05) (Table 3**)**. Specifically, each one-year increase in age was associated with an average 3.0% reduction in the speed of BP goal achievement. Patients with a history of hypertension had a speed of achievement that was 0.542 times that of patients without a history of hypertension. For each 1 mmHg increase in postoperative initial SBP, the speed of achievement decreased by an average of 2.4%. For each 1-point increase in postoperative pain score, the speed of achievement decreased by an average of 22.8%.

**Table 3:** Multivariate Cox regression analysis of factors influencing time to BP goal achievement.

Variable	*β* coefficient	*HR*	95% *CI*	Wald *χ²*	*P*
Age	−0.031	0.970	(0.948, 0.993)	6.523	0.011
History of hypertension	−0.611	0.542	(0.352, 0.834)	7.874	0.005
Postoperative initial SBP	−0.024	0.976	(0.961, 0.992)	8.912	0.003
Postoperative pain score	−0.259	0.772	(0.652, 0.913)	9.415	0.002

BP, Blood pressure; SBP, Systolic blood pressure; HR, Hazard ratio; CI, Confidence interval.

### 3.2 Results of developing the refined nursing monitoring protocol

#### 3.2.1 Indicator selection and weight determination based on literature and Delphi expert consultation

After two rounds of Delphi expert consultation, expert opinions converged. Kendall's W coefficients were 0.423 and 0.486 for the two rounds, respectively (both *P* < 0.01). By calculating the x¯ ± s of the experts' importance ratings for each indicator at all levels, the final indicators for the refined nursing monitoring protocol and their weights were determined. The first-level indicators and their weights were: risk stratification management (0.30), refined BP monitoring (0.35), standardized pain management (0.20), and multidisciplinary collaboration (0.15).

#### 3.2.2 Framework and core content of the refined nursing monitoring protocol

Based on the above research, the final refined nursing monitoring protocol for post-TEVAR TBAD patients includes the following core content:

1. Risk stratification management protocol: A quantitative scoring system was used for risk stratification (Table 4), and tiered management is implemented.
2. Refined BP monitoring protocol

**Table 4:** Risk stratification criteria for post-TEVAR BP management.

Risk level	Score	Criteria
Low risk	0–1 point	Possesses none or only one risk factor
Medium risk	2–3 points	Possesses 2–3 risk factors
High risk	≥ 4 points	Possesses 4 or more risk factors

Each risk factor counts as 1 point: ① Age ≥60 years; ② History of hypertension; ③ Postoperative initial SBP ≥ 140 mmHg; ④ Postoperative pain score ≥ 4 points. TEVAR, thoracic endovascular aortic repair; BP, blood pressure; SBP, systolic blood pressure.

All BP monitoring was performed by registered nurses who have received specialized training in post-TEVAR management. Invasive arterial BP monitoring was managed by ICU specialized nurses or senior responsible nurses to ensure proper catheter maintenance and accurate waveform interpretation. High-risk patients were monitored every 15–30 min within the first 2 h postoperatively, every 30 min from 2 to 6 h, and hourly from 6 to 24 h. Medium-risk patients were monitored every 30 min within the first 2 h postoperatively, and every 1–2 h from 2 to 24 h. Low-risk patients followed the standard postoperative monitoring protocol. For all patients whose BP remained unachieved after 24 h, the monitoring frequency corresponding to their current risk level was maintained until goals were met. Priority for invasive arterial BP monitoring was given to high-risk patients, patients requiring vasoactive drugs, and patients with non-invasive BP fluctuations >20 mmHg/h. When monitoring detected BP outside the target range (SBP > 120 mmHg or <100 mmHg), the following protocol was initiated: ① BP was re-measured within 5 min to rule out human or equipment error. ② The responsible nurse immediately assessed the patient for pain, anxiety, urine output, and vasoactive drug infusion status. For hypertension, adequate analgesia/sedation was ensured. For hypotension, potential active bleeding or cardiac dysfunction was investigated. ③ If the abnormal reading persisted for ≥2 consecutive measurements, or if a single reading showed SBP > 160 mmHg or <90 mmHg, the attending physician or on-call physician was immediately notified. ④ If accompanied by neurological symptoms such as nausea, vomiting, restlessness, or altered level of consciousness, the patient was considered a medical emergency, prompting immediate activation of the rapid response team. ⑤ Antihypertensive or vasopressor medication was adjusted as prescribed. After medication adjustment, monitoring frequency was increased to every 15 min until BP remained stable within the target range for at least 1 h, then reverted to the original frequency.

3. Standardized pain management protocol

The foundational analgesic was intravenous acetaminophen (1 g), administered every 6 h. For patients with moderate pain (NRS score of 4–6), non-steroidal anti-inflammatory drugs (NSAIDs) (e.g., parecoxib sodium, flurbiprofen axetil) were added. For patients with severe pain (NRS score > 7), a low-dose opioid (e.g., hydromorphone, sufentanil) was added.

Individualized alternative regimen was initiated when contraindications existed to drugs in the standard regimen. If acetaminophen was contraindicated, an NSAID (if no contraindication) or a low-dose opioid was considered as the foundational analgesic. If NSAIDs were contraindicated, a weak opioid was considered. Simultaneously, non-pharmacological therapies such as massage, cold/heat application, relaxation training, and distraction techniques were initiated. For severe pain patients requiring extreme caution with or intolerant to opioids, multimodal analgesia was immediately initiated (e.g., nerve blocks, patient-controlled intravenous analgesia). Any medication adjustment was jointly decided by the anesthesia pain physician, clinical pharmacist, and attending physician to ensure medication safety.

4. Multidisciplinary collaboration (MDT)

An MDT team was established, comprising vascular surgeons, anesthesia pain physicians, clinical pharmacists, and specialized nurses. Joint rounds were conducted daily in the morning, focusing on high-risk patients. Consultation was initiated immediately if a patient's BP remained outside the warning range for over 30 min without improvement.

## 4 Discussion

This study systematically analyzed the factors influencing the time to achieve early BP goals in TBAD patients after TEVAR and developed a targeted refined nursing monitoring protocol, providing an important reference for clinical practice. The results confirmed that advanced age, history of hypertension, higher postoperative initial SBP, and higher pain scores were independent risk factors for prolonging this time.

While confirming traditional risk factors such as advanced age and hypertension history, this study extends beyond the limitations of previous research. Compared to the study on postoperative hypertension by Franklin et al. (26), this study further quantified the specific impact of each factor on the time to BP goal achievement. Mancia et al. (27) similarly highlighted the association between BP variability and long-term outcomes, whereas this study primarily focused on the time to early postoperative BP goal achievement, a time-sensitive indicator directly impacting the early recovery process. Although Vasan et al. (28) observed an association between pain and BP fluctuations in their study, they did not explore this relationship in depth. Through multivariate analysis, this study is the first to confirm that postoperative pain is an independent risk factor for delayed BP goal achievement after TEVAR, and it works in conjunction with other factors to form an interconnected pathophysiological basis.

The four independent factors identified in this study are not isolated but synergistically amplify the sympathetic nervous system output, collectively constituting a unified pathophysiological network that delays BP goal achievement. Advanced age (associated with blunted baroreceptor reflex and reduced vascular compliance) and a long-standing history of hypertension (linked to RAAS activation and vascular remodeling) together establish the pathological foundation for heightened sympathetic reactivity and impaired BP autoregulation (29, 30). These lead to an upward shift in the threshold for cardiovascular autonomic regulation and decreased systemic stability, impairing appropriate negative feedback response to BP fluctuations. Postoperative initial high SBP is the body’s direct physiological response to the significant stress of surgical trauma, marking a burst release of catecholamines (e.g., norepinephrine) (31). In this cohort, intraoperative blood loss was moderate (median 340 mL) and not the primary driver of hypotension. Rather, greater surgical trauma (potentially correlated with higher blood loss) might contribute to a more pronounced stress response, leading to higher initial postoperative SBP, which aligns with our finding that elevated initial SBP is a risk factor for delayed goal achievement. This phase sets a high initial starting point for postoperative BP control, significantly increasing the difficulty of subsequent management. Simultaneously, nociceptive stimuli from postoperative pain ascend via the spinal cord, directly exciting cardiovascular centers such as the rostral ventrolateral medulla. This drives sympathetic outflow, sustains catecholamine secretion, elevates BP and heart rate, and forms a positive feedback loop with the aforementioned factors (32, 33). Therefore, the multimodal analgesia emphasized in our protocol works by interrupting this neuroendocrine vicious cycle, creating a favorable physiological milieu for BP control (18). Therefore, the multimodal analgesia emphasized in this protocol essentially works by interrupting this neuroendocrine vicious cycle. Furthermore, In the present study, we observed that the median time to achieve blood pressure goals was 9.5 h. This finding suggests that achieving stringent early postoperative blood pressure control following thoracic endovascular aortic repair (TEVAR) in current clinical practice can be challenging, indicating room for optimization. This further underscores the necessity of developing a systematic, risk-stratified, and refined monitoring protocol to more effectively manage key postoperative factors such as sympathetic overactivation, initial hypertension, and pain, thereby potentially shortening the time to goal achievement and enhancing overall management efficiency.

This study transcends the limitations of single-factor analysis by integrating the four independent factors into a pathophysiological network model for the first time and, based on this, constructing a refined nursing monitoring protocol centered on risk stratification. This protocol elevates analgesia to a key component of BP management, shifting from passive monitoring to active intervention. It provides clinical practice with a structured, actionable blueprint. By accurately identifying high-risk patients and implementing multimodal analgesia and tiered monitoring, it can effectively optimize nursing resource allocation, shorten the time to BP goal achievement, and holds significant value for improving patient prognosis and promoting homogeneity and precision in post-TEVAR nursing care (34).

Although the refined nursing monitoring protocol developed in this study possesses important clinical translational value, enabling prospective risk estimation through quantitative scoring and ensuring the timeliness and scientific rationale of BP and pain management decisions, reflecting a patient-outcome-centered integrated care philosophy, it still has limitations. First, As a single-center retrospective study, while it can reveal associations, it cannot establish causality, and the generalizability of the conclusions is limited. Second, the risk stratification scoring system developed from this single-center data requires external validation through multicenter, prospective studies to confirm its predictive efficacy and generalizability. Third, to obtain complete data on the time to blood pressure goal achievement, we excluded patients who died or were discharged within 24 h postoperatively. This exclusion may have introduced selection bias, as those experiencing early death or unstable discharge were likely the very population with the most challenging blood pressure control or hemodynamic instability. Consequently, our study findings may be more applicable to the patient cohort that survived the initial 24-hour critical period, and the overall estimates may be somewhat optimistic. Fourth, this study focused on the process indicator of time to BP goal achievement. Although postoperative complications were recorded, their association with this time metric was not analyzed due to sample size constraints and the retrospective design. This relationship warrants exploration in future larger-scale studies. Furthermore, the study has two data-level shortcomings. First, the lack of uniformity in BP monitoring methods introduces inherent accuracy differences that may potentially affect the precision of the recorded time to achievement. Second, reliance on nursing documentation in a retrospective study means variations in recording quality and standardization among different nurses, and despite cross-checking, recording bias cannot be completely eliminated.

Future research should validate the effectiveness of this protocol through multicenter, prospective cohort studies. Building on this, efforts should promote the deeper development of postoperative BP management towards digitization and personalization. Establishing an intelligent nursing monitoring system based on real-time data, integrating wearable devices, invasive/non-invasive BP monitors, and EMR data, and utilizing artificial intelligence algorithms to achieve real-time early warning of BP trends and dynamic assessment of intervention effects could enhance management efficiency (1, 17). Concurrently, in-depth exploration of individualized BP management strategies should be pursued, extending beyond exploring medication combinations based on pathophysiological subtypes to studying optimal BP target ranges and precise non-pharmacological intervention regimens for patients with different risk stratifications and complications, thereby advancing post-TEVAR BP management to a new stage of greater precision and proactivity.

In summary, advanced age, history of hypertension, higher postoperative initial SBP, and higher postoperative pain scores are independent risk factors for delayed achievement of BP goals after TEVAR. The refined nursing monitoring protocol developed based on these findings demonstrates strong scientific rigor, systematic structure, and clinical operability. It can provide an evidence-based justification and practical reference for standardizing and optimizing postoperative management in TBAD patients. The impact of implementing this protocol on clinical outcomes requires further prospective validation.

## Data Availability

The original contributions presented in the study are included in the article/Supplementary Material, further inquiries can be directed to the corresponding author.
